# Towards a more comprehensive assessment of cardiovascular fitness - magnetic resonance augmented cardiopulmonary exercise testing (MR-CPEX)

**DOI:** 10.1186/1532-429X-15-S1-P58

**Published:** 2013-01-30

**Authors:** Bejal Pandya, Grzegorz T Kowalik, Daniel S Knight, Oliver Tann, Graham Derrick, Vivek Muthurangu

**Affiliations:** 1 Cardiac MR, Great Ormond Street Hospital and Institute of Cardiovascular Sciences, University College London, London, UK

## Background

Assessment of exercise intolerance is important in patients with cardiovascular disease. Traditionally, this is achieved by measuring maximum VO2 during cardio-pulmonary exercise testing (CPEX). However, this cannot discriminate between cardiac output and tissue extraction problems. A better approach may be to assess VO2 and cardiac output simultaneously (which also allows calculation of tissue extraction). Thus, we developed MR augmented CPEX in which real- time PCMR is performed at the same time as respiratory gas analysis. The purpose of this study was to validate this novel technology.

## Methods

Ten volunteers underwent MR-CPEX in a 1.5T scanner using a ramped protocol on an MR compatible Ergometer. All volunteers exercised till exhaustion and the total test period was 9 minutes. Expired gases and respiratory flow data were collected with a calibrated MR compatible respiratory analysis system. Using this data continuous VO2, VCO2 and Ve were calculated for the whole test period. Aortic flow was measured continuously during the test period using real-time UNFOLD-SENSE spiral PCMR (spatial resolution: 2.5x2.5 mm, temporal resolution: 30 ms, 16000 frames). Flow data was segmented using a semi-automated technique to calculate cardiac output during exercise. Cardiac output and VO2 were used to calculate arterio-venous oxygen content gradient (tissue oxygen extraction). All volunteers also underwent traditional bicycle CPEX for comparison.

## Results

**Figure 1:**
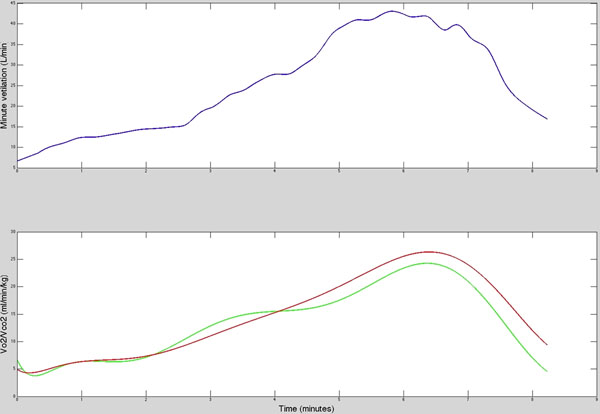
Ventilation (top) and simultaneous VO2 (green) /VCO2 (red) curves during MR-CPEX in a volunteer achieving their anaerobic threshold.

**Figure 2:**
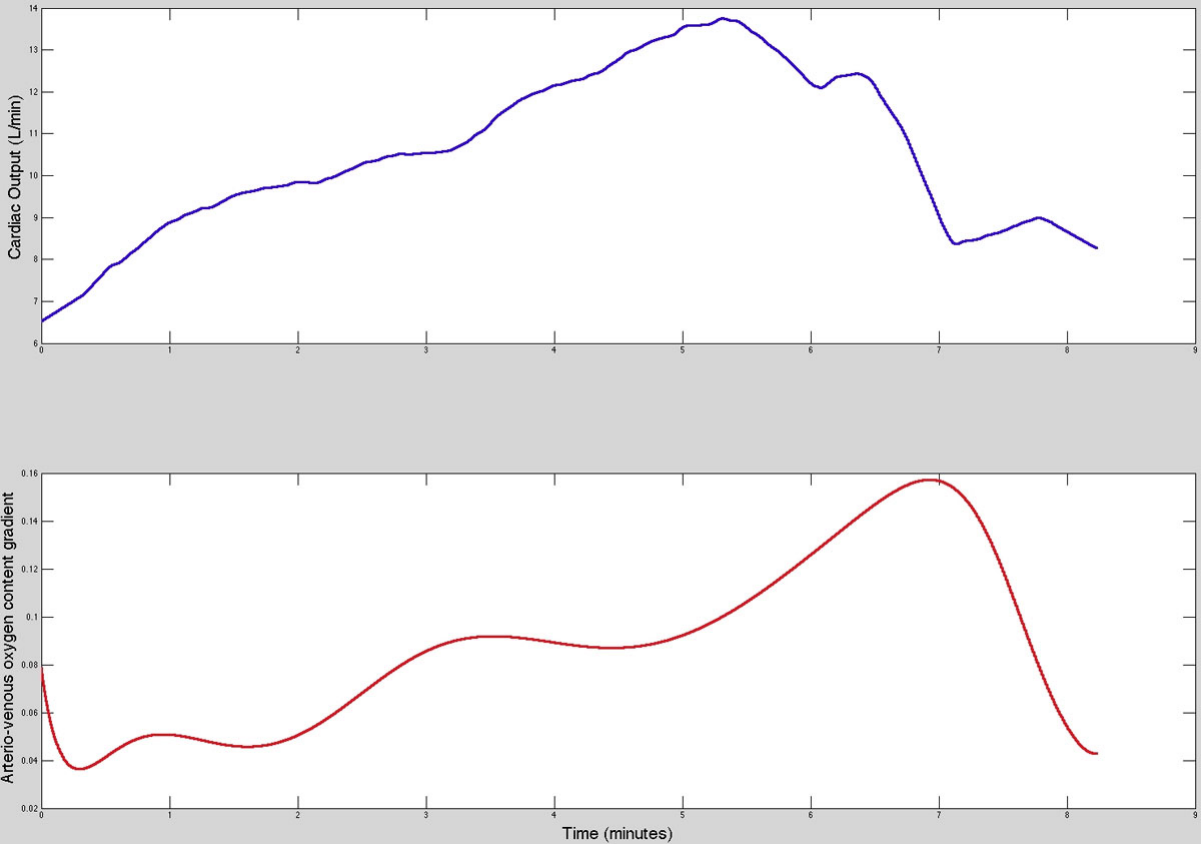
Arterio-venous Oxygen content gradient (top) and Cardiac Output (bottom) during MR-CPEX.

MR augmented CPEX was successful in all volunteers with 40% of participants reaching their anaerobic threshold. The maximum workload reached during MR and conventional CPEX was strongly correlated. (r=0.76). Mean peak VO2 during MR-CPEX was 19.3±5.1ml/min/kg and peak VCO2 was 19.6±5.5ml /min/kg. There was an excellent correlation between MR-CPEX peak VO2 and conventional CPEX (r=0.84). During MR-CPEX, mean heart rate rose from 76±14 to 151±25 bpm, with no change in stroke volume. This resulted in mean cardiac output increasing from 3.2±0.5 l/min/m2 to 6.6±1.2 l/min/m2. Mean peak arterio-venous oxygen gradient calculated from the cardiac output and VO2 during MR-CPEX was 12ml O2 per 100ml of blood. Representative ventilation, cardiac output and tissue arterio-venous oxygen gradient curves are shown in figures 1 &2.

## Conclusions

This study shows that MR-CPEX is a viable technique that can provide a comprehensive assessment of all the components of exercise physiology. During MR-CPEX only submaximal exercise is possible, both due to movement limitation and lack of stroke volume augmentation in the supine position. Nevertheless, there was as strong correlation between traditional CPEX and MR-CPEX. This implies that MR-CPEX does measure useful parameters that are linked to maximal exercise. We believe that the ability to fully measure the cardio-pulmonary and peripheral response to exercise will allow better assessment of exercise intolerance in many cardiac diseases.

## Funding

British Heart Foundation Project Grant. Dr Vivek Muthurangu and Dr Bejal Pandya.

